# Early Sensory Loss Alters the Dendritic Branching and Spine Density of Supragranular Pyramidal Neurons in Rodent Primary Sensory Cortices

**DOI:** 10.3389/fncir.2019.00061

**Published:** 2019-09-25

**Authors:** Tamar Macharadze, Eike Budinger, Michael Brosch, Henning Scheich, Frank W. Ohl, Julia U. Henschke

**Affiliations:** ^1^Department Systems Physiology of Learning, Leibniz Institute for Neurobiology, Magdeburg, Germany; ^2^Clinic for Anesthesiology and Intensive Care Medicine, Otto von Guericke University Hospital, Magdeburg, Germany; ^3^Center for Behavioral Brain Sciences, Magdeburg, Germany; ^4^Special Lab Primate Neurobiology, Leibniz Institute for Neurobiology, Magdeburg, Germany; ^5^Emeritus Group Lifelong Learning, Leibniz Institute for Neurobiology, Magdeburg, Germany; ^6^Institute for Biology, Otto von Guericke University, Magdeburg, Germany; ^7^Institute of Cognitive Neurology and Dementia Research (IKND), Otto von Guericke University, Magdeburg, Germany

**Keywords:** cortex, crossmodal, deprivation, development, Golgi, integration, intercortical, multisensory

## Abstract

Multisensory integration in primary auditory (A1), visual (V1), and somatosensory cortex (S1) is substantially mediated by their direct interconnections and by thalamic inputs across the sensory modalities. We have previously shown in rodents (Mongolian gerbils) that during postnatal development, the anatomical and functional strengths of these crossmodal and also of sensory matched connections are determined by early auditory, somatosensory, and visual experience. Because supragranular layer III pyramidal neurons are major targets of corticocortical and thalamocortical connections, we investigated in this follow-up study how the loss of early sensory experience changes their dendritic morphology. Gerbils were sensory deprived early in development by either bilateral sciatic nerve transection at postnatal day (P) 5, ototoxic inner hair cell damage at P10, or eye enucleation at P10. Sholl and branch order analyses of Golgi-stained layer III pyramidal neurons at P28, which demarcates the end of the sensory critical period in this species, revealed that visual and somatosensory deprivation leads to a general increase of apical and basal dendritic branching in A1, V1, and S1. In contrast, dendritic branching, particularly of apical dendrites, decreased in all three areas following auditory deprivation. Generally, the number of spines, and consequently spine density, along the apical and basal dendrites decreased in both sensory deprived and non-deprived cortical areas. Therefore, we conclude that the loss of early sensory experience induces a refinement of corticocortical crossmodal and other cortical and thalamic connections by pruning of dendritic spines at the end of the critical period. Based on present and previous own results and on findings from the literature, we propose a scenario for multisensory development following early sensory loss.

## Introduction

There is a growing number of evidences in various species that multisensory integration in first-level sensory cortices, such as primary auditory (A1), visual (V1), and somatosensory (S1) areas, is substantially mediated by direct connections between these cortical regions and by crossmodal thalamic inputs (rodents: Henschke et al., 2015; non-human primates: Cappe et al., 2009; humans: Ro et al., 2013; for comparison of animal species: Meredith and Lomber, 2017). Functionally speaking, direct anatomical connections across the sensory modalities at this early level of cortical processing enables short neuronal latencies (rodents: Iurilli et al., 2012; Sieben et al., 2013; non-human primates: Brosch et al., 2005; Wang et al., 2008; humans: Sperdin et al., 2009; Raij et al., 2010) and fast entrainment of ongoing cortical activity to non-matched sensory stimuli (rodents: Sieben et al., 2013; non-human primates: Lakatos et al., 2007; humans: Mercier et al., 2013). At the behavioral level, this functional-anatomical substrate may be instrumental for improving sensory performance of individuals (crossmodal facilitation effect: Welsh and Warren, 1986; Stein and Meredith, 1993; Calvert et al., 2001), as seen in shorter reaction times to crossmodal stimuli compared to unimodal stimuli (rodents: Sakata et al., 2004; Gleiss and Kayser, 2012; humans: Gielen et al., 1983; Molholm et al., 2002;Noesselt et al., 2010).

In adult humans and animals, sensory loss leads to functional improvements of the remaining senses (for review, see Lomber et al., 2010; Merabet and Pascual-Leone, 2010; Frasnelli et al., 2011; Renier et al., 2014; Teichert and Bolz, 2017). Several neuronal mechanisms have been suggested to drive this crossmodal (compensatory) plasticity, such as the formation of new pathways or unmasking, strengthening, and remodeling of existing connections during development (for review, see Bavelier and Neville, 2002; Feldman and Brecht, 2005; Barnes and Finnerty, 2010; Kupers and Ptito, 2014; Meredith et al., 2017). In line with these proposed mechanisms, we have recently shown by means of retrograde tracer experiments in Mongolian gerbils, that early auditory, visual, and somatosensory deprivation indeed leads to an increase of multisensory corticocortical (intercortical) and thalamocortical connections of A1, V1, and S1 at the end of the sensory critical period (postnatal day P28) in this rodent species (Henschke et al., 2018a). Sensory matched thalamocortical connections were also increased. Results of simultaneously performed immunohistological analyses of expression levels of markers for neurogenesis (doublecortin), apoptosis (cysteinyl-aspartate specific protease 3), and axonal plasticity (growth associated protein 43) suggested to us that this increase in anatomical connectivity is mediated by local axonal reorganization processes, i.e., *via* sprouting of crossmodally-projecting axons in the sensory deprived but also spared (non-deprived) cortical areas (Henschke et al., 2018a). Consistent with these anatomical findings, *in vivo* single-photon emission computed tomography of cerebral blood flow revealed a higher functional connectivity specifically between the primary sensory areas. However, regardless of the increased crossmodal anatomical and functional connectivity, sensory deprived animals show an overall decrease in the level of neuronal activity in primary sensory areas in response to sensory stimulation by both their own (matched) as well as by other (non-matched) modalities (Henschke et al., 2018a). Consistent with this finding, neonatal (transient) whisker trimming leads to smaller sensory-evoked electrical responses (from both tactile and visual stimulation) within supragranular layers of S1 in P19–22 rats (Sieben et al., 2015).

In the present follow-up study, we investigated the possible underlying anatomical substrate leading to the increased multisensory anatomical and functional connectivity but decreased stimulus-evoked activity. We did this at the single-cell level using a random sample of Golgi-impregnated supragranular layer III pyramidal neurons in A1, V1, and S1 of normal and early sensory deprived Mongolian gerbils. Because the dendrites of layer III pyramidal neurons are the main targets of intercortical (mainly to apical dendrites) and thalamocortical (mainly to basal dendrites) projections (for review, see Nieuwenhuys, 1994; Bannister, 2005; Winer, 2011; Budinger and Kanold, 2018), we investigated whether their dendritic morphology, including spine number and density, is specifically altered due to the loss of early sensory experience.

Previous studies using similar approaches (Golgi-stain) but across a variety of species, developmental stages, and deprivation paradigms have produced conflicting results. For example, adult cats, which were ototoxically deafened when young, showed an increased spine density of supragranular pyramidal neurons in the matching (deprived) primary sensory area A1 (Clemo et al., 2017). Similarly, following 1 month of mouse whisker trimming after birth, the spine density of basal dendrites in layer IV of the barrel cortex increased (P30 and P60; Chen et al., 2015). In contrast to this increase in spine density within the deprived modality, neonatal destruction of one cochlea leads to a decrease of spines on basal dendrites of supragranular pyramidal neurons in contralateral A1 of young adult rabbits (P60; McMullen and Glaser, 1988). At an even earlier postnatal time point, neonatal eye enucleation in rabbits causes a spine decrease on apical dendrites in supragranular layers of V1 at P30 (Globus and Scheibel, 1967). In line with these results, enucleation of newborn mice reduces the number of spines on the apical dendrites of pyramidal neuron in layers III (and V) in V1 (but not A1), which is significant already at P30 and lasts until P180 (Heumann and Rabinowicz, 1982). Besides the latter study, there are no further studies investigating the effects of early sensory deprivation on the morphology of supragranular neurons in areas of the non-deprived (spared) modalities. Thus, in order to bridge the gap between previous findings in differently aged animals and between various (deprived and spared) sensory systems, our present study investigates the effects of early sensory loss at an important developmental time point (P28, end of the critical sensory period in gerbils), at the level of three primary sensory cortices (A1, V1, S1), using three different deprivation paradigms (ototoxic inner hair damage, eye enucleation, sciatic nerve transection), and investigating the resulting effects on the sensory deprived as well as spared cortices simultaneously.

## Materials and Methods

### Experimental Animals

Experiments were performed on 12 Mongolian gerbils (*Meriones unguiculatus*). Animals were of both genders and P28 at the end of the experiment. For each group (including control) three gerbils were used. Animals were housed together with their littermates and mother in standard laboratory cages (Tecniplast, Italy; Eurostandard Type IV, 598 × 380 × 200 mm) in air-conditioned rooms (average temperature 22°C, 12 h light-dark cycle) with water and food available *ad libitum*. All experiments were performed according to the NIH Guide for the Care and Use of Laboratory animals (2011) and the Directive of the European Communities Parliament and Council on the protection of animals used for scientific purposes (2010/63/EU) and were approved by the animal care committee of Sachsen-Anhalt, Germany (number of proposal for animal experimentation: 42502-2-1324 LIN).

The Mongolian gerbil is a small, agile, and robust desert rodent (Milne-Edwards, 1867), which is easy to breed and handle in captivity (Schwentker, 1963). It originated from the steppes of Mongolia and belongs to the *muridae* family, which also includes mouse and rat; subfamily *gerbillinae* (Musser and Carleton, 2005). A DNA sequence analysis of mitochondrial genes suggests a split with lineage leading to mice and rats approximately 13 million years ago (Chevret and Dobigny, 2005). A recent *de novo* sequencing and initial annotation of the Mongolian gerbil genome revealed that the gerbil shares 87.2% of its genes with mouse and 82.8% with human, while 84.7% are shared between mouse and human (Zorio et al., 2019). The ancestors of nearly all Mongolian gerbils, commonly used in laboratories, went through a genetic bottleneck in the 1950s; thus, they are inbred like most laboratory mouse and rat strains (Stuermer et al., 2003).

Mongolian gerbils have a lot of sensory characteristics that make them an ideal model for various kinds of sensory research (Schwentker, 1963; Budinger and Scheich, 2009; Zorio et al., 2019) such as our own present and previous studies. In contrast to nocturnal rats and mice, gerbils are primarily diurnal (Thiessen and Yahr, 1977). They have good visual capabilities including blue and green color vision (Govardovskii et al., 1992; Jacobs and Deegan, 1994) as well as superior visual acuity and photopic vision compared to rats and mice (Baker and Emerson, 1983; Yang et al., 2015). This may be based on the relatively high percentage of cone photoreceptors in the gerbil’s retina, which is more analogous to the human retina (Govardovskii et al., 1992; Bytyqi and Layer, 2005). Gerbils also have a human-like sensitivity to low-frequency sounds (2–4 kHz; Lay, 1972; Ryan, 1976), whereas rats and mice are more sensitive to very high frequencies (for comparison of species see: Heffner et al., 2001). In humans, this low-frequency sensitivity matches the acoustic spectrum of speech; in gerbils, it matches the spectrum of their communication signals produced by vocalization and hind paw drumming (Finck and Goehl, 1968). Latter is a conspicuous warning behavior that gerbils use in certain situations, for example, when they detect potential predators (first mentioned by Thomas, 1908). Hence, gerbils well perceive touch (Thiessen and Yahr, 1977; Cabana et al., 1993) as well as have a well-developed olfaction typical for rodents (Pettijohn and Paterson, 1982; Clark et al., 1986). Mongolian gerbils show anatomical, physiological, and behavioral evidence for crossmodal interactions, even at the level of primary sensory cortices (Cahill et al., 1996; Budinger and Scheich, 2009; Kobayasi et al., 2013; Mowery et al., 2016; Henschke et al., 2018a), which seem to be similarly organized in other rodent species (Campi et al., 2010; Henschke et al., 2015; Meredith and Lomber, 2017).

The Mongolian gerbil is also a favorable species for research on development and aging (Vincent et al., 1980; Cheal, 1986; Henschke et al., 2018a,b). At birth, Mongolian gerbils are deaf and blind (Souter et al., 1997) but show first sensorimotor reflexes (Cabana et al., 1993). Gerbils develop slower than rats and mice (Schwentker, 1963), particularly during the activation of the different sensory systems. While there is a partial temporal overlap between the onset of hearing and vision in mice and rats, this is not the case in gerbils enabling a precise investigation of the different sensory influences during development with respect to their onset. In mice, ears open at around P10–12 (Ehret, 1976) and eyes around P12–15 (Fuller and Wimer, 1966). In gerbils, external ear canal and middle ear cavity are completely open at P14 (Finck et al., 1972) and eyes open between P16 and 20 (average P18.5 ± 1.2 days: Wilkinson, 1986; Mowery et al., 2016). Young gerbils are weaned around P28; they are sexually mature 2–3 months after birth and live to an average of 3.5 years, i.e., longer than mice (Vincent et al., 1980; Cheal, 1986).

### Early Sensory Deprivation

*Visual deprivation* was performed at P10 by bilateral eye enucleation (Chabot et al., 2007; Henschke et al., 2018a). For enucleation, the eyelid of the anesthetized animal was carefully opened and the eyeball was displaced from its socket using round forceps. The optic nerve and ophthalmic artery were then clamped with fine forceps for 2 min and, thereafter, the optic nerve was cut and the eyeball removed. The orbital cavity was filled with absorbable styptic gelatine sponge (Gelastypt, Sanofi-Aventis; Germany) and the eyelid was closed with surgical silk (Johnson and Johnson, NJ, USA).

*Somatosensory deprivation* was performed at P5 by a bilateral transection of the sciatic nerve of the hindlimb (Wall and Cusick, 1986; Henschke et al., 2018a). This particular somatosensory deprivation was chosen based on previous studies demonstrating multisensory connections preferentially for the hindlimb area (HL) of the gerbil’s S1 (Budinger et al., 2006; Henschke et al., 2015) and the consequent finding that auditory and visual deprivation effects the connectivity and activity of S1-HL neurons more than of the other S1 fields (Henschke et al., 2018a). We already speculated previously that the particular role of S1-HL in multisensory processing may be related to the hind paw drumming warning behavior of gerbils, namely that this behavior leads to substrate vibrations that are picked up by the animals with their hind paws and then, the vibratory information is integrated with auditory and visual information at the cortical level (Budinger et al., 2006). Littermates were separated from their mother; then, each animal was anesthetized with isoflurane (4 vol%) and positioned on its side. The skin around the upper femoral joint was locally disinfected, anesthetized, and finally incised along the femur. By displacement of the outer femoral muscles the sciatic nerve became visible, was carefully separated from the surrounding tissue and vessels, and finally transected. The surgical opening was treated with an anti-inflammatory ointment and sutured. The animal was put back with its littermates and 1 h later with its mother. The maturation of each animal was carefully monitored and the transection of the sciatic nerve resulted in no major motor consequences except a partial overflexion of the hind paw metatarsophalangeal joints.

*Auditory deprivation* was performed by bilateral ototoxic inner hair cell damage (Heydt et al., 2004; Henschke et al., 2018a) at P10. For deafening, the skin behind the pinna of the anesthetized animals was locally disinfected and incised. A small hole was drilled into the tympanic cavity and 0.5 μl Gentamycin (1% in distilled water, 0.0025% EDTA, 0.00008% sodium bisulfite; Sigma-Aldrich, Germany) was applied directly onto the round window using a microliter syringe (Hamilton, Switzerland). Thereafter, the tympanic cavity was closed with bone wax and the skin incision was treated with an anti-inflammatory ointment and sutured. The animal was put back with its littermates and 1 h later with its mother. At P28, deafness of animals was validated by a lack of the startle response as described previously (Bhattacharya et al., 2017). Briefly, a startle stimulus (50 ms, 120 dB) was delivered to the gerbil in a startle-box system (TSE Systems GmbH, Germany) with or without preceding prepulse stimulus (30 ms, 100 ms before the startle stimulus) at eight different intensities (73–94 dB, 3 dB increments) on a 70 dB white noise background. After habituation to the box (3 min), two startle trials were followed in pseudo-random order by 10 startle trials and five trials at each of the prepulse intensities with stochastically varied intertrial intervals (5–30 s). The maximal startle amplitude (if present at all) was measured by a sensor platform. Only animals, which did not show any sign of acoustic responses, i.e., which were obviously completely deaf, were used for this study.

### Golgi Preparations

**Figure 1:**
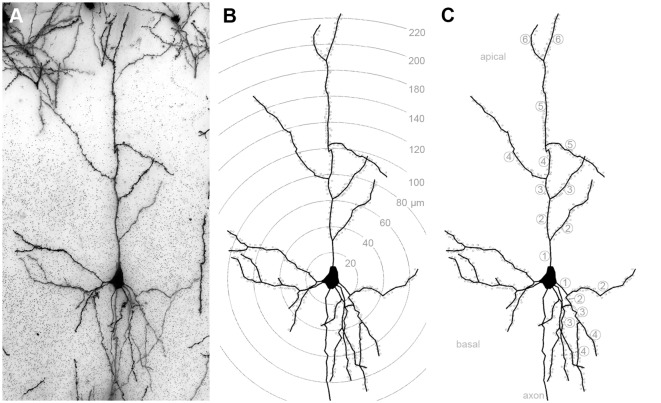
**(A)** Golgi-stained layer III pyramidal neuron in A1. **(B)** Same neuron reconstructed by means of the Neurolucida software (MicroBrightField, Europe). Shells centered around the soma illustrate the 20 μm segments used for Sholl analysis of dendritic branches and spines (dots). **(C)** Same reconstructed neuron; numbers indicate branch orders of the apical and of one basal dendrite as used for branch order analysis.

**Figure 2:**
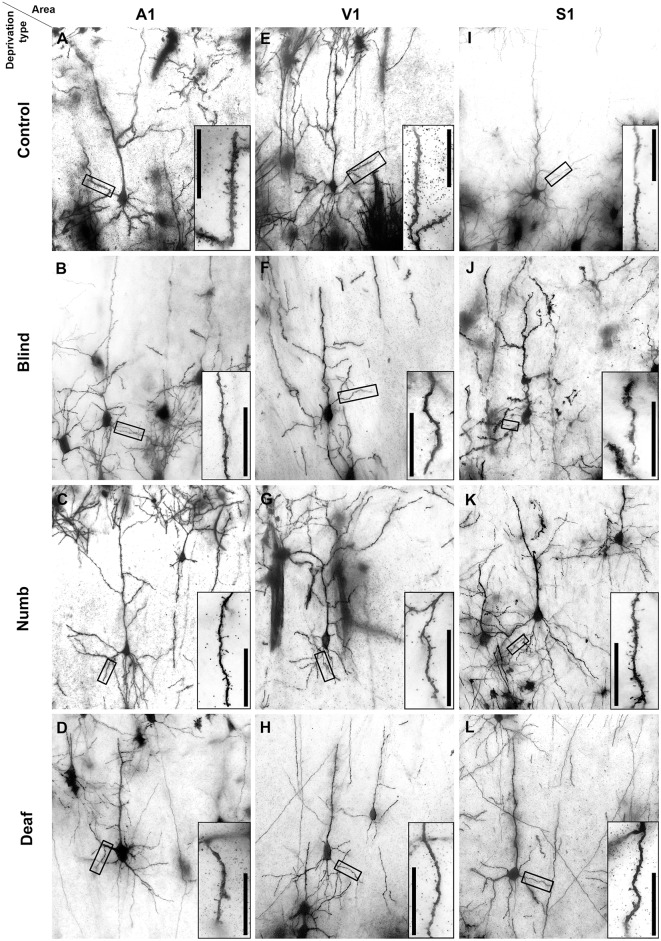
Golgi-stained layer III pyramidal neurons in A1 **(A–D)**, V1 **(E–H)**, and S1-HL **(I–L)** of normal P28 animals **(A,E,I)** and animals following early visual **(B,F,J)**, somatosensory **(C,G,K)**, and auditory deprivation **(D,H,L)**. Insets show close-ups of enframed dendritic branches (always basal dendrite, 2nd order). Scale bars = 20 μm.

At P28, animals were sacrificed by an overdose of pentobarbital (20 mg/100 g body weight, i.p.; Sigma-Aldrich). Brains were extracted and incubated in the dark for 14 days at room temperature with 50 ml of a Golgi–Cox solution as described by Glaser and Van der Loos, 1981 and as used previously (Mylius et al., 2013; Figures 1A, 2). Specifically, a 5% solution of potassium dichromate (Merck, Germany) and a 5% solution of mercuric chloride (Merck) were mixed at a ratio of 1:1. Next, 100 ml of this solution was added to 140 ml of a 5% solution of potassium chromate (Merck), which was previously diluted in distilled water (1:2.5). The final Golgi-Cox solution was kept in the dark for at least 5 days at room temperature before brains were placed into the solution for impregnation. After impregnation, brains were dehydrated in a graded series of ethanol (3 h in 50%, 24 h in 70%, 24 h in 96%, and 24 h in 100% ethanol at 4°C), treated in a mixture of ethanol (100%) and anhydrous diethylether (1:1) for 4 h, and embedded in a graded series of celloidin (2 days in 2%, 3 days in 4%, and 4 days in 8% celloidin). Next, brain-containing celloidin blocks were formed and dried in a desiccator for several days under exposure to phosphorus pentoxide (Merck) and polymerized and hardened under exposure to chloroform (Merck). Blocks were stored in 70% ethanol at 4°C until sectioning. On a sliding microtome (Microm, Germany), brains were cut into serial sections of 150 μm thickness in coronal planes. The orientation of the cutting plane was the same as used in the gerbil stereotaxic atlas (Radtke-Schuller et al., 2016), i.e., perpendicular to the horizontal line connecting the highest points of the cerebrum and cerebellum. The sections were collected in 70% ethanol and rinsed in distilled water. They were then treated in an alkaline ammonia solution (1:1 in distilled water) for 45 min in the dark and in 0.5% phenylen-diamine (Sigma-Aldrich) for an additional 5 min. Following repeated rinsing in distilled water, the staining was developed in 1% dectol (Kodak, Germany) for 2 min and fixed in 5% tetenal (Calbe Fotochemie, Germany) for 5 min. Finally, sections were rapidly dehydrated in a graded series of ethanol and xylol (Roth, Germany) and mounted between two coverslips using Merckoglas mounting media (Merck).

### Data Analysis

The sections were thoroughly inspected using a standard brightfield microscope (Leica DMRX, Germany). Regions of interest (A1, V1, S1-HL) were identified using stereotaxic information from the gerbil brain atlas (Radtke-Schuller et al., 2016) and from previous publications about the Golgi architecture of the gerbil’s auditory system (Budinger et al., 2013; Mylius et al., 2013). Specifically, S1-HL was identified according to its relative location to internal brain structures like the primary motor cortex (M1), hippocampus, and corpus callosum. It is the most rostral and medial part of S1, adjacent to M1, latter having a very distinctive lamination pattern (nearly absent layer IV). The rostrocaudal extent of S1 is about 2 mm, ranging in frontal sections from the transition between the genu and truncus of the corpus callosum until the appearance of the dorsal hippocampus. In order to be absolutely sure about their location in S1-HL, we always chose neurons from the middle range of this area, which corresponds to the crossing of the anterior commissure (around atlas plate 23; Radtke-Schuller et al., 2016), for analysis. A1 extends rostrocaudally from the appearance of the ventral hippocampus until the disappearance of the dorsal lateral geniculate body and ventrodorsally about 2 mm dorsal from the temporal pole (maximal lateral extension of the brain at this level). We chose neurons from the center of A1, which corresponds approximately to plate 31 of the atlas. V1 is the largest primary sensory field and comprises a monocular (V1M) and a binocular part (V1B). We chose neurons from the transition zone between both areas from cortical sections, where hemispheres were just no longer connected by the corpus callosum (atlas plate 34).

In P28 gerbils, layer III of A1 is located 200–400 μm under the cortical surface; in S1-HL and V1 200–350 μm (Henschke et al., 2018a). Using Golgi material, a shrinkage of 10%–20% compared to paraformaldehyde fixated material has to be considered; thus, we also orientated at the “normalized” distance from the pia related to the cortical thickness: given the cortical thickness as 100%, layer III covers the distance 25% to maximal 40% from the pia in all three areas. For identification of layer III pyramidal neurons also other cytoarchitectural features were considered such as the absence of pyramidal cell somata in layer IV and the smaller size of pyramidal neurons in layer II compared to layer III (for review, see Nieuwenhuys, 1994; Bannister, 2005; Winer, 2011; Budinger and Kanold, 2018). The different ages of the animals used in the current (P28) and previous studies (P120; Budinger et al., 2013; Mylius et al., 2013; Radtke-Schuller et al., 2016) had not to be considered because the brain size and in particular the cortical thickness does not significantly change from P28 towards P120 animals (Wilkinson, 1986; Henschke et al., 2018a,b).

The morphology of 144 layer III pyramidal neurons (12 neurons *per* area and deprivation type, including controls; equally distributed over the left and right hemispheres and all animals of the respective experimental group) was reconstructed by means of a *camera lucida* system connected to the microscope (NeuroLucida v. 11.07; MicroBrightField, Europe) and using 63× magnification. After reconstruction, morphometric parameters of their dendrites were analyzed using the Sholl and branch order analysis tools of NeuroExplorer (v. 11.03; MicroBrightField). Sholl analysis revealed the number of intersections between dendrites and Sholl segment borders (20 μm distance) and dendritic lengths within Sholl segments (20 μm radius) as a measure of dendritic branching (Sholl, 1953). Also, the number and density (number/dendritic length) of spines within these Sholl segments was analyzed (Figure 1B). The length of dendrites, number of spines, and spine density per branch order in a dendritic tree was evaluated by the branch order analysis (Figure 1C). Branch order analysis provides information about the morphology of entire branches of a given order within a dendritic tree. Thus, it is not as sensitive to spatial parameters as the Sholl analysis because of the wider spatial range covered by each individual branch compared to the smaller (20 μm) Sholl segments (Sholl, 1953).

Statistical analysis, including Shapiro–Wilk-test for testing normal distribution as well as two-tailed, unpaired Student’s *t*-test (when values were normally distributed) and Mann–Whitney-*U*-test (when values were not normally distributed) for group comparisons, was performed using Microsoft Excel (v. 15.40 for Mac) and the Xlstat application (xlstat.com). Images were taken with a digital camera (Optronix Macrofire, CA, USA) mounted on the microscope. Illustrations were arranged using Photoshop CS4 (v. 11.0.2 for Mac).

## Results

Examples of Golgi-impregnated supragranular pyramidal neurons in layer III of A1, V1, and S1-HL in normal (control) as well as visual (blind), somatosensory (numb), and auditory (deaf) deprived animals are given in Figure 2.

### Comparison Across Areas in Normal Animals

**Figure 3:**
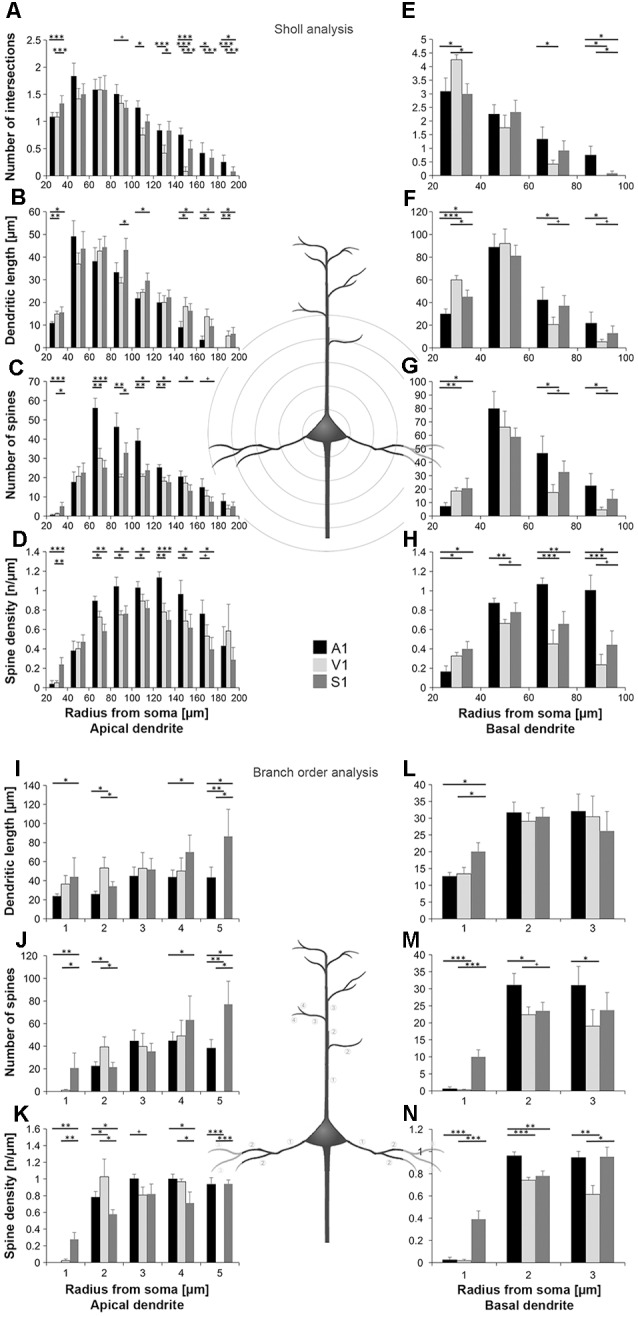
Comparison of dendritic morphology of layer III pyramidal neurons in A1, V1, and S1-HL using Sholl **(A–H)** and branch order **(I–N)** analysis. Depicted are the numbers of intersections, dendritic lengths, numbers of spines, and spine densities for apical (left) and basal (right) dendrites. Values are mean ± 1 standard error of the mean (SEM); comparisons are A1 vs. S1 (upper line of crossbars), A1 vs. V1 (middle line), and V1 vs. S1 (lower line); ^+^*p* ≤ 0.10, *p ≤ 0.05, **p ≤ 0.01, ***p ≤ 0.001, Student’s *t*-test (for normally distributed values) and Mann–Whitney-*U*-test (for not normally distributed values); *n* = 12 cells for each group.

Before investigating the effects of early sensory deprivations, we compared in detail the morphology of layer III pyramidal neurons across A1 (Figure 2A), V1 (Figure 2E), and S1-HL (Figure 2I) in normal P28 animals. This is the first comparison of that kind in a rodent species and will shed light on the anatomical basis of putative areal-specific and branch type-dependent mechanisms of dendritic integration processes in these areas. As evident by the Sholl and branch order analyses, there are several differences in the branching patterns and spine distributions between A1, V1, and S1-HL neurons (Figure 3).

Using Sholl analysis, branching patterns of *apical dendrites* differed mainly in their very proximal and their distal parts. Close to the soma (20–40 μm), the number of intersections and dendritic lengths were highest for S1 neurons (Figures 3A,B). More distant from the soma (>100 μm), the number of intersections was usually highest for A1 neurons and lowest for V1 neurons (Figure 3A). In the range of 80–140 μm apart from the soma, dendritic lengths within Sholl segments were longest for S1-HL neurons; at very distal parts (>140 μm), they were shortest for A1 neurons (Figure 3B). Spine numbers and densities of A1, V1, and S1-HL neurons differed in close proximity to the soma (20–40 μm), showing most spines and highest spine density in S1-HL, as well as in the range 60–180 μm apart from the soma, where spine numbers and density were always highest for A1 neurons (Figures 3C,D).

Branch order analysis of apical dendrites revealed longer higher-order (4–5) branches of S1-HL neurons than of A1 and V1 neurons (Figure 3I). Most spines were found on 1st order branches of S1-HL and on 2nd order branches of V1 neurons as well as on higher-order (4–5) branches of S1-HL neurons (Figure 3J). At 1st order branches, spine densities were highest for S1-HL neurons; at 2nd order branches for V1, and at 3rd and 4th order branches for A1 (Figure 3K), which corresponds to the results of the Sholl analysis (Figures 3C,D).

**Figure 4:**
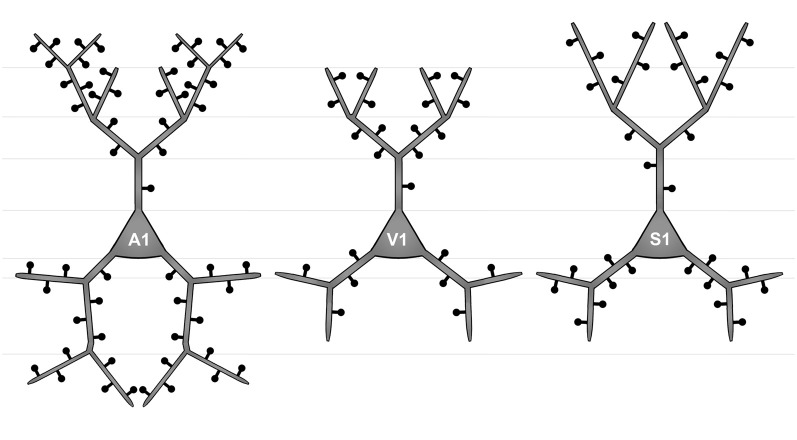
Simplified schematic illustrating the morphological differences between layer III pyramidal neurons in A1 (left), V1 (middle), and S1-HL (right) in P28 gerbils with normal sensory experience.

Taken Sholl and branch order analysis of apical dendrites together (Figure 4), S1-HL layer III pyramidal neurons had longest proximal (1st order) dendritic aspects bearing most spines and having highest spine density. S1-HL neurons had also longest higher-order dendritic branches. V1 neurons had also many spines and a high spine density at proximal dendrites (2nd order) but least branching (intersections) at higher orders. A1 neurons had generally most widely branched dendritic arbors (intersections) with highest spine densities.

Differences in the morphology of *basal dendrites* were most prominent in their very proximal (<40 μm, Sholl analysis) and in distal parts (>60 μm). Proximal dendritic aspects were longest in V1 neurons (Figure 3F), which also had most intersections (Figure 3E), and in S1-HL neurons (Figure 3L). Spine number and spine density were highest in S1-HL neurons as seen in the Sholl (20–40 μm, Figures 3G,H) and branch order analysis (1st order, Figures 3M,N). Distal dendritic aspects were longest in A1 neurons and had most intersections with Sholl segments 60–100 μm apart from the soma (Figures 3E,F). In a similar range, spine number and spine density were highest for A1 and lowest for V1 neurons (Figures 3G,H). Again, this was also evident in the branch order analysis (2nd and 3rd order, Figures 3M,N).

Taken Sholl and branch order analysis of basal dendrites together (Figure 4), V1 and S1-HL layer III pyramidal neurons had longer proximal dendritic aspects than A1 neurons; however, most spines and the highest spine density were at very proximal S1-HL branches. A1 neurons had longer and more widely branched distal dendrites, where these neurons had also more spines and a higher spine density than V1 and S1-HL neurons.

In our sample of neurons from the center of areas A1, V1, and S1-HL in normal animals we did not see any significant differences in the morphology of neurons neither with respect to their rostrocaudal location within a given area nor with respect to their distance from the cortical surface. This held also true for neurons of the deprived animals.

### Effects of Early Sensory Loss

#### Primary Auditory Cortex (A1)

**Figure 5:**
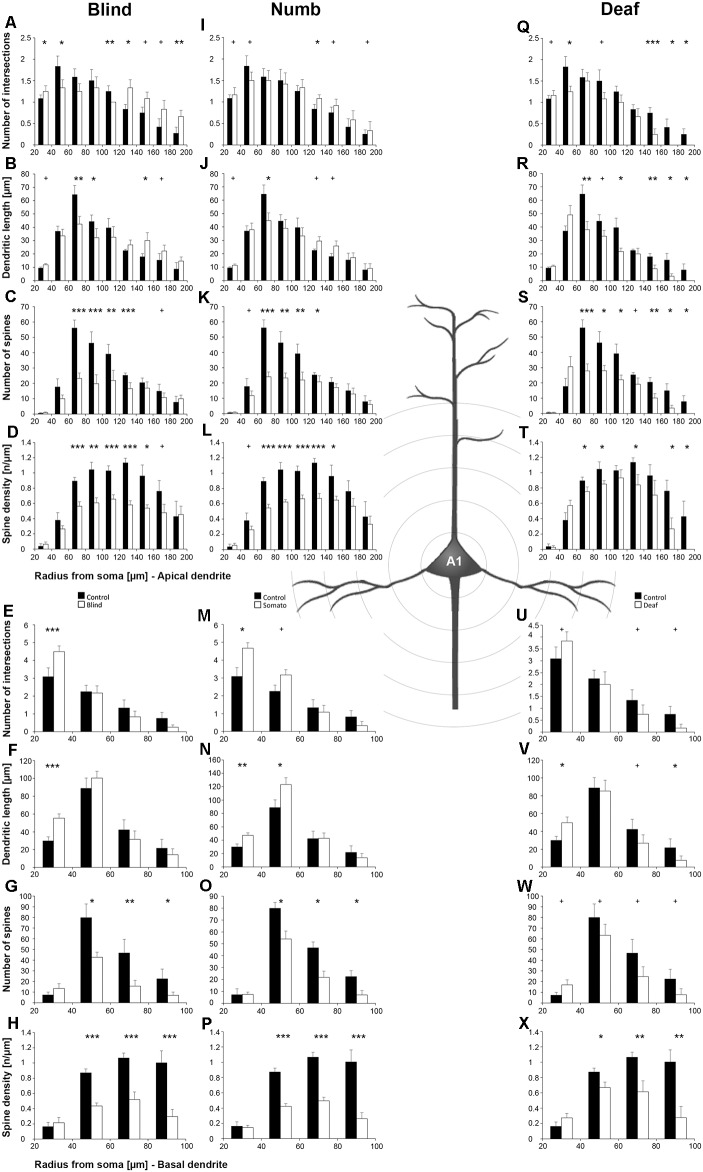
Sholl analysis of layer III pyramidal neurons in A1 following visual **(A-H)**, somatosensory **(I-Q)**, and auditory **(R-X)** deprivation. Depicted are the numbers of intersections, dendritic lengths, numbers of spines, and spine densities for apical (top half) and basal (bottom half) dendrites compared to control animals. Values are mean ± 1 SEM; ^+^*p* ≤ 0.10, *p ≤ 0.05, **p ≤ 0.01, ***p ≤ 0.001; Student’s *t*-test (for normally distributed values) and Mann–Whitney-*U*-test (for not normally distributed values); *n* = 12 cells for each group.

**Figure 6:**
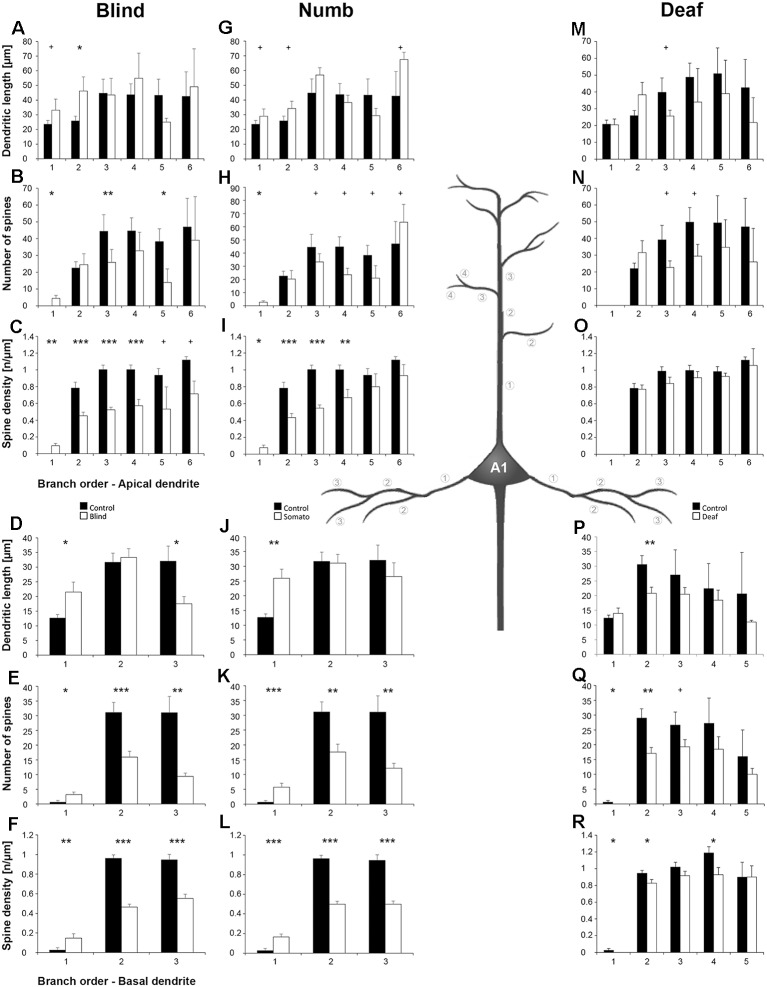
Branch order analysis of layer III pyramidal neurons in A1 following visual **(A-F)**, somatosensory **(G-M)**, and auditory **(N-R)** deprivation. Depicted are the dendritic lengths, numbers of spines, and spine densities for apical (top half) and basal (bottom half) dendrites compared to control animals. Values are mean ± 1 SEM; ^+^*p* ≤ 0.10, *p ≤ 0.05, **p ≤ 0.01, ***p ≤ 0.001; Student’s *t*-test (for normally distributed values) and Mann–Whitney-*U*-test (for not normally distributed values); *n* = 12 cells for each group.

Statistical results of Sholl and branch order analysis for A1 following early visual, somatosensory, and auditory deprivation are depicted in Figures 5 and 6.

Compared to control animals, *visual deprivation* led to an increase of dendritic branching of layer III pyramidal neurons in A1 at very proximal aspects (20–40 μm) of their apical and basal dendrites and at distal aspects (>120 μm) of their apical dendrites as seen in the increase of the intersections with and dendritic lengths within Sholl segments (Figures 5A,B,E,F). In the range 40–120 μm apart from the soma, dendritic branching of apical dendrites was decreased (Figures 5A,B). Branch order analysis supported these changes of branching patterns between control and deprived animals only to some part, i.e., mainly for the proximal dendrites (Figures 6A,D), which might be due to the lower spatial resolution of branch order analysis compared to Sholl analysis (see “Materials and Methods” section). However, visual deprivation resulted in a general decrease of the spine number and spine density of apical and basal dendrites in A1, which was evident in both Sholl (Figures 5C,D,G,H) and branch order analysis (Figures 6B,C,E,F).

*Somatosensory deprivation* caused similar changes of branching patterns like visual deprivation, namely an increase in dendritic branching of apical (Figures 5I,J) and basal dendrites (Figures 5M,N, 6J) at proximal and distal aspects as well as a slight decrease of branching in middle aspects of apical dendrites (Figures 5I,J). Interestingly, this middle range (60–120 μm apart from the soma) coincides with the apical region, where A1 neurons of normal animals already have the highest overall spine number and density (Figure 3C), and which may be therefore subject of different compensation mechanisms than the other dendritic aspects. However, again as for visual deprivation, the spine number and spine density at apical (Figures 5K,L, 6H,I) and basal dendrites (Figures 5O,P, 6K,L) was drastically decreased following somatosensory deprivation.

In contrast to visual and somatosensory deprivations, *auditory deprivation* (i.e., deprivation of the matched modality) led in A1 to a general decrease in dendritic branching of apical (Figures 5Q,R, 6M) and basal dendrites (Figures 5U,V, 6P). Only very proximal aspects of apical (Figure 5Q) and basal dendrites (Figures 5U,V) became longer and more branched. Nevertheless, still the number of spines at apical (Figures 5T, 6N) and basal (Figures 5W, 6Q) dendrites decreased in such large numbers that the spine density significantly decreased (Figures 5T,X, 6R) despite the shorter dendritic lengths.

#### Primary Visual Cortex (V1)

**Figure 7:**
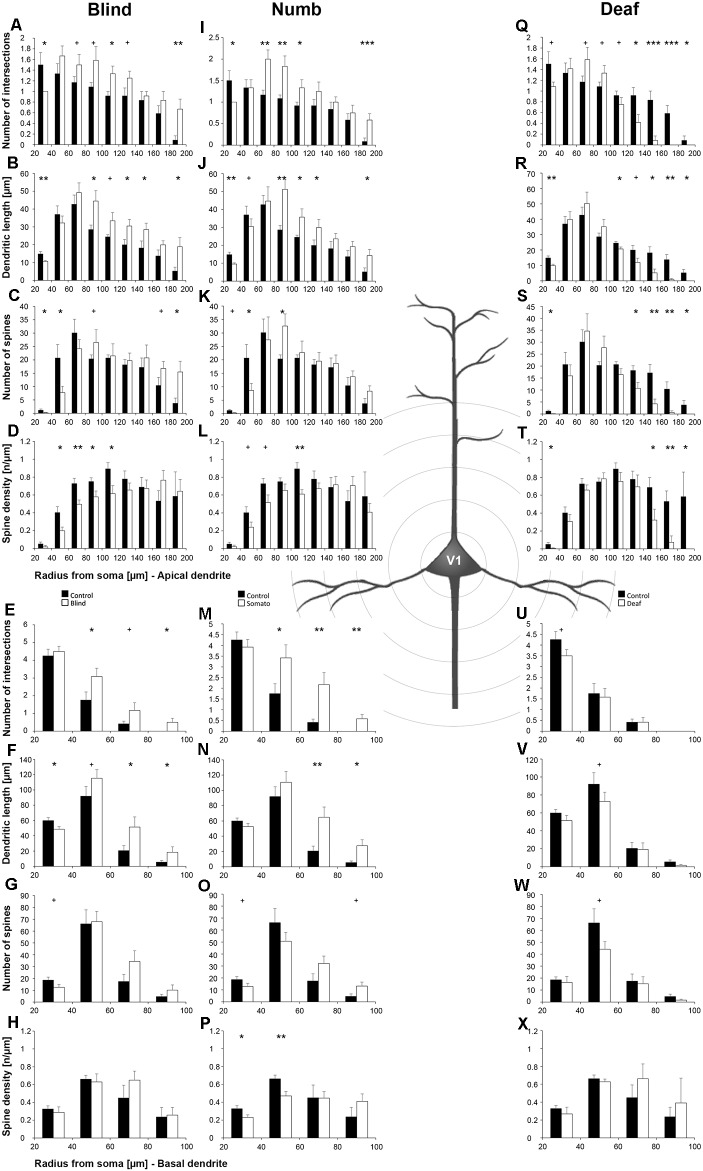
Sholl analysis of layer III pyramidal neurons in V1 following visual, somatosensory, and auditory deprivation. All conventions as in Figure 5.

**Figure 8:**
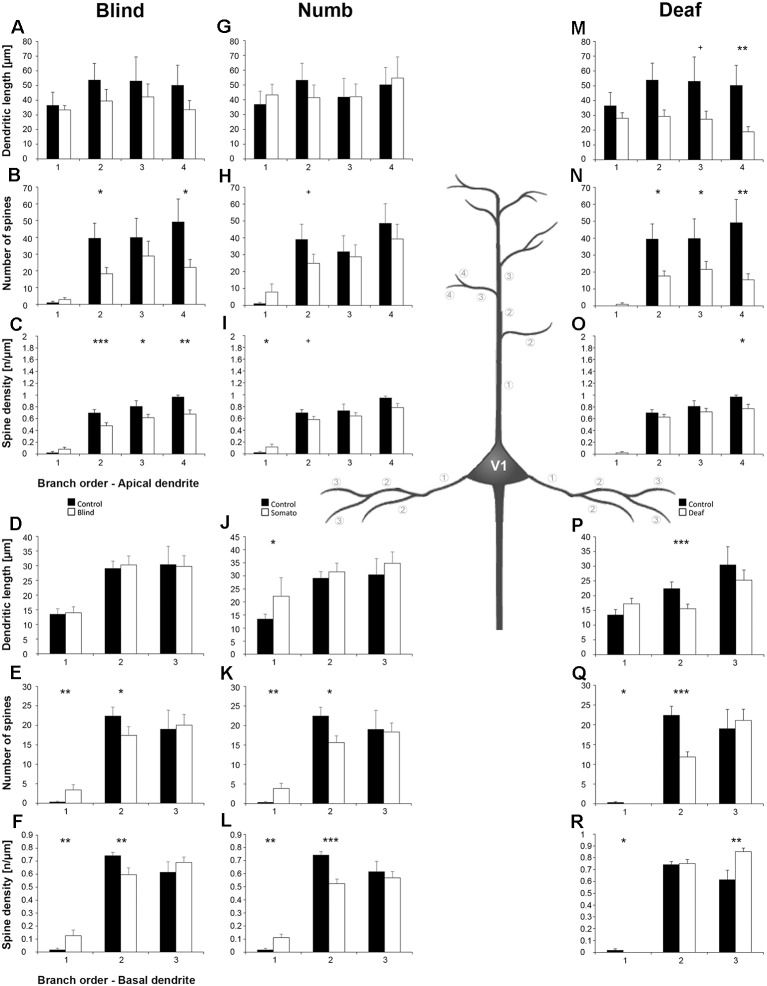
Branch order analysis of layer III pyramidal neurons in V1 following visual, somatosensory, and auditory deprivation. All conventions as in Figure 6.

Statistical results of Sholl and branch order analysis for V1 following early visual, somatosensory, and auditory deprivation are depicted in Figures 7 and 8.

Deprivation of the *visual* (i.e., matched) modality resulted in an increase of the dendritic branching (intersections and dendritic lengths) of apical (Figures 7A,B) and basal dendrites (Figures 7E,F) in V1 of deprived animals compared to controls; only very proximal dendritic aspects became shorter and less branched (Sholl analysis: 20–40 μm, Figures 7A,B; branch order analysis: 1st order, Figure 7F). This is contrary to A1, where the deprivation of the matched modality led to a general decrease of dendritic branching except for very proximal aspects (see above). However, like for A1, the number of spines and the spine density along apical and basal dendrites of layer III pyramidal neurons in V1 was largely decreased (Figures 7C,D,G,H, 8B,C,E,F).

Also, *somatosensory deprivation* led to a general increase of dendritic branching of apical and basal dendrites (Figures 7I,J,M,N) but with the opposite effect on very proximal apical dendritic aspects (Scholl analysis: 20–40 μm, Figures 7I,J). Though with some exceptions, the number, and even more so the density (Figures 7L,M, 8I,L), of spines decreased.

Similar to the deprivation effects observed in A1 (see above), *auditory deprivation* led to a decrease of the dendritic branching particularly of apical (Figures 7Q,R, 8M) but also basal dendrites in V1 (Figures 7U,V, 8P). Thus, auditory deprivation itself and not the deprivation of the matched cortical area caused a decrease of the dendritic branching. Like in A1, also the spine number and density particularly for apical dendrites (Figures 7S,T, 8R,N) decreased.

#### Primary Somatosensory Cortex (S1, Hindlimb Area)

**Figure 9:**
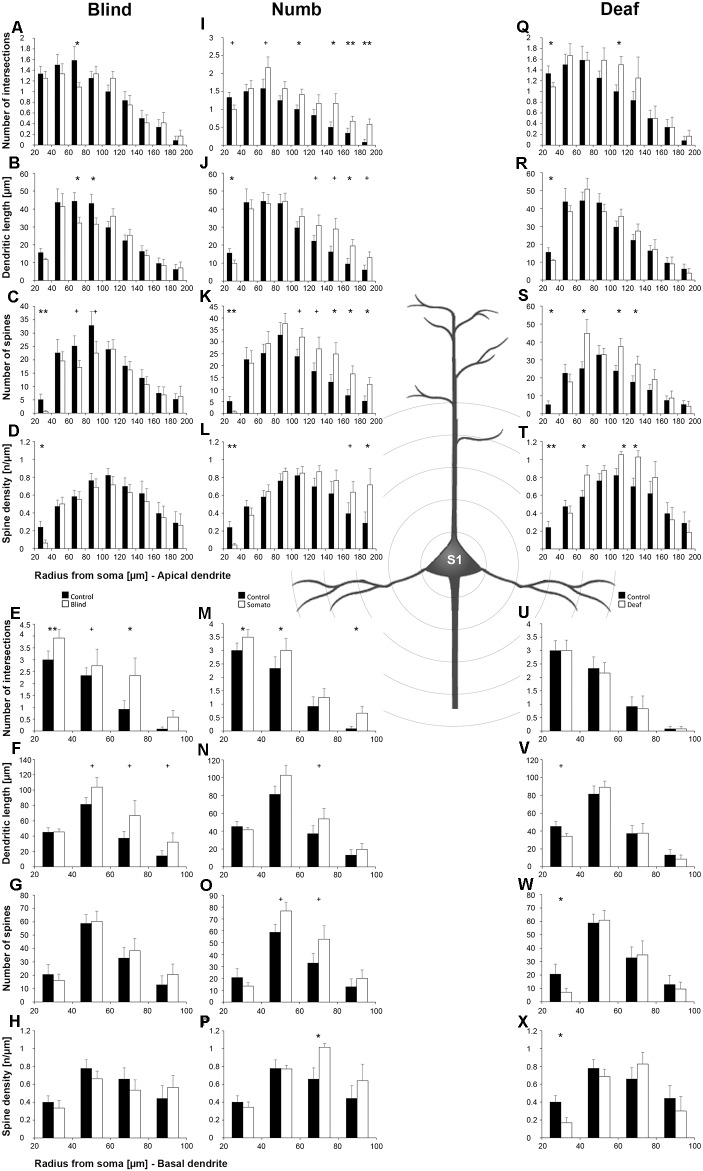
Sholl analysis of layer III pyramidal neurons in S1-HL following visual, somatosensory, and auditory deprivation. All conventions as in Figure 5.

**Figure 10:**
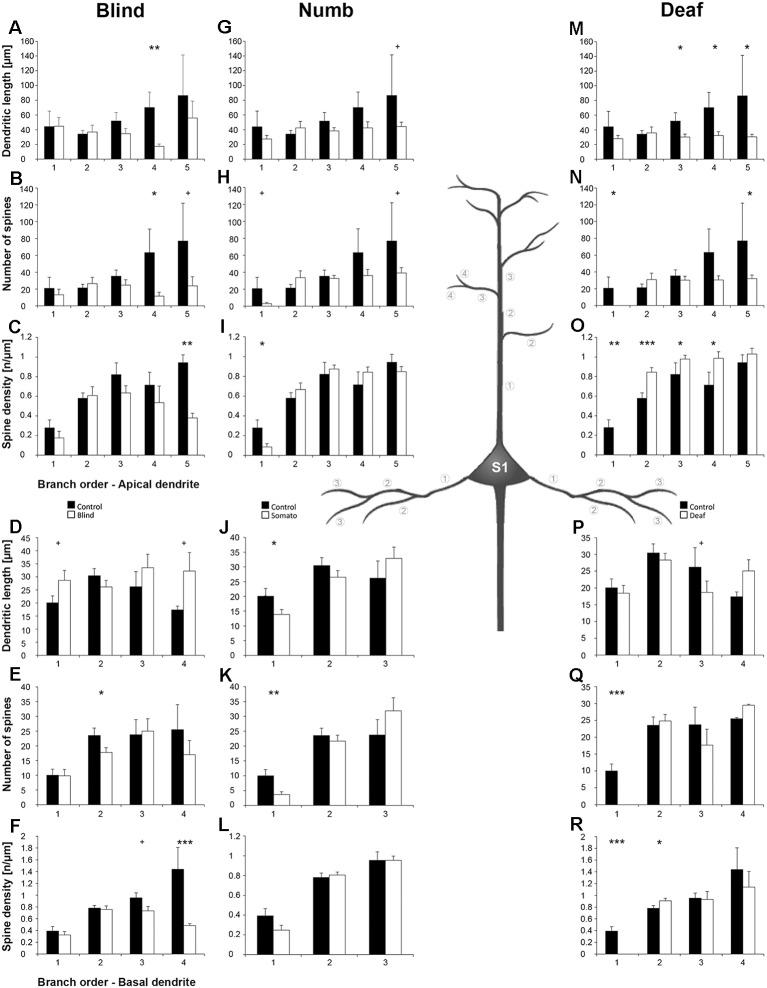
Branch order analysis of layer III pyramidal neurons in S1-HL following visual, somatosensory, and auditory deprivation. All conventions as in Figure 6.

Statistical results of Sholl and branch order analysis for S1-HL following early visual, somatosensory, and auditory deprivation are depicted in Figures 9 and 10.

*Visual deprivation* tended to cause an increase of the dendritic branching in mainly basal dendrites of S1-HL layer III pyramidal neurons (Figures 9E,F, 10D). However, in the Sholl analysis for apical dendrites (Figures 9A,B), the dendritic length 60–100 μm away from the soma was decreased (similar to A1, see above). Spine number and density slightly decreased or stayed rather constant (Figures 9C,D,G,H, 10B,C,E,F).

*Somatosensory deprivation* (i.e., deprivation of the matched modality) led to an overall increase of the dendritic branching (Figures 9I,J,O,P) in deprived animals compared to controls. In contrast to all other deprivation types and areas described so far, the number of spines along apical and basal dendrites increased, which was most evident in the Sholl analysis (Figures 9K,O). Consequently, the spine density did not decrease as seen in all other deprivation types and areas described so far, but rather stayed constant or even increased (Figures 9L,P, 10I,L). Notable exceptions were dendritic regions very proximal to the soma, where spine number and density decreased (Sholl analysis: 20–40 μm, Figures 9K,L; branch order analysis: 1st order, Figures 10I,K).

With some exceptions, *auditory deprivation* caused a slight decrease of the dendritic branching, most obviously seen in the branch order analysis of apical dendrites (Figure 10M); similar to the effects seen from auditory deprivation in both A1 and V1. The spine number, in particular of apical dendrites, usually increased as well as the spine density (Figures 9S,T, 10O,P). Notable exceptions were the very proximal regions of both apical and basal dendrites, where spine number and density decreased (Scholl analysis: 20–40 μm, Figures 9S,T,W,X; branch order analysis: 1st order, Figures 10N,O,Q,R).

### Summary of Results

There are several differences in the branching patterns and spine distributions between A1, V1, and S1-HL layer III pyramidal neurons in normal P28 animals (Figure 4). A1 neurons had generally most widely branched apical and basal dendrites (in particular distal aspects) with highest spine densities (spines per given dendritic length). S1-HL and V1 neurons had longer proximal dendritic aspects (apical and basal) than A1 neurons with highest spine densities at S1-HL dendrites. S1-HL neurons had longest higher-order apical branches, A1 neurons had longest higher-order basal branches.

**Figure 11:**
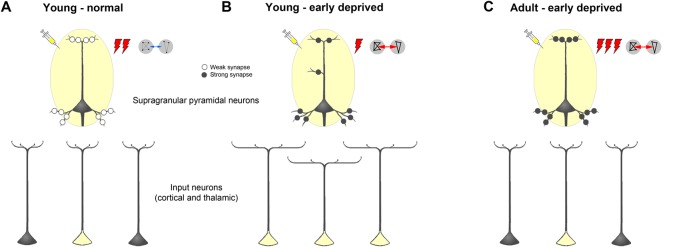
Schematic summarizing alterations of dendritic morphology of supragranular pyramidal neurons in primary sensory cortices due to early sensory deprivation and their presumptive functional consequences as derived from own present and previous studies as well as other literature (for references see text). **(A,B)** Compared to young individuals, which experienced a normal sensory environment until the end of the sensory critical period, supragranular pyramidal neurons in sensory deprived individuals show a wider dendritic branching but fewer spines and lower spine density. In young deprived individuals, axonal branches of input neurons are also wider, leading to more labeled cells after retrograde tracer injections. Due to less synaptic contacts stimulus-evoked activity is lower, but due to synaptic strengthening functional connectivity is higher. **(C)** In early deprived adult individuals, dendritic branches of supragranular pyramidal neurons, as well as axonal branches of input neurons are pruned back to normal, but new and/or already existing synaptic contacts are formed and strengthened. This leads after retrograde tracer injections to the same number of labeled input neurons, as in normal adult individuals but to a higher activity, particularly to non-matched sensory stimuli in spared areas, and to a higher functional connectivity between primary sensory cortical areas.

Early somatosensory, auditory, and visual deprivation led to several morphological changes of these neurons (Figures 11A,B). With few exceptions particularly concerning very proximal dendritic aspects, branching of apical and basal dendrites, as seen by the number of intersections and dendritic lengths, was increased in all three areas following visual and somatosensory deprivation. In contrast, dendritic branching, particularly of apical dendrites, was decreased in all three areas following auditory deprivation. Both effects were most noticeable in V1 and A1 and less pronounced in S1-HL.

Most importantly, after sensory deprivation the number of spines along the apical and basal dendrites was reduced and the spine density decreased. These effects were most evident in A1 and V1 and less pronounced in S1-HL. As a noteworthy exception, spine number and density of apical dendrites increased in S1-HL following auditory and somatosensory deprivation.

## Discussion

### Effects of Early Sensory Loss on the Morphology of Supragranular Pyramidal Neurons

The aim of the present study was to investigate, on the cellular level, the possible anatomical basis of the counterintuitive finding that the loss of early sensory experience leads to increased anatomical and functional connectivity between primary sensory areas but also decreased stimulus-driven activity in both sensory deprived and non-deprived (spared) regions at the end of the critical sensory period (Henschke et al., 2018a; see also: Sieben et al., 2015).

Our results provide evidence that the decreased stimulus-driven activity may be explained by the pruning of dendritic spines at the apical and basal dendrites of the main target cells of these intercortical (and of thalamocortical) multisensory connections, namely the supragranular layer III pyramidal neurons (for review, see Nieuwenhuys, 1994; Bannister, 2005; Winer, 2011; Budinger and Kanold, 2018). That is, although there are more widely branched axons of the crossmodally projecting (and other sensory matched) input neurons (leading to an increased number of retrogradely labeled cells in sensory cortex and thalamus after cortical tracer injections), the number of actual synaptic contacts is drastically reduced due to post-synaptic pruning of spines (Figures 11A,B). Such pruning was also described in V1 of early blinded rabbits and P30 mice (Globus and Scheibel, 1967; Heumann and Rabinowicz, 1982) as well as in S1 of transiently (P0–7) whisker-trimmed P30 mice (Tjia et al., 2017). As a consequence of this reduction in synaptic contacts, the overall activity upon sensory stimulation (as seen, for the example, by electrophysiological recordings and regional cerebral blood flow; Sieben et al., 2015; Henschke et al., 2018a) decreases. In addition to the synaptic pruning, increased local inhibition might contribute to this activity decrease as seen by the strengthening of transmission from inhibitory to excitatory V1 neurons in layer IV of visually deprived mice (Nahmani and Turrigiano, 2014).

On the other hand, we recently showed (Henschke et al., 2018a) that there is an increased functional connectivity, i.e., an increased correlation of—although overall reduced—activity between sensory cortical areas in deprived animals. This may be explained by a higher effectiveness of the remaining synaptic contacts due to synaptic strengthening at the molecular (e.g., increased glutamate receptor density; Bridi et al., 2018) and ultrastructural (e.g., larger spine heads and post-synaptic densities: Vees et al., 1998) levels. In other words, due to fewer synaptic contacts stimulus-evoked activity is lower, but due to synaptic strengthening functional connectivity is higher.

Our results suggest, that this scenario is largely applicable for both, sensory deprived and spared cortical areas. The early loss of sensory input, regardless of being from the matched (deprived) or non-matched (spared) modality, seems to cause a general pruning of spines in supragranular pyramidal neurons in primary sensory cortices at the end of the sensory critical period. Using a random sample of Golgi-impregnated neurons, as we and previous authors did, one can not specify whether these neurons receive indeed multisensory or only sensory matched inputs. In gerbils, the probability that they are (directly or *via* local intracortical circuits) influenced by other modalities is about 1:4 based on electrophysiological (27% of investigated neurons in A1 are modulated by visual stimulation; Kobayasi et al., 2013) and anatomical studies (18% of the anatomical input to A1 comes from non-auditory sources; Budinger and Scheich, 2009). In other species, this probability may be similar or even higher (ratio of multisensory neurons in A1, ferret 15%–32%: Bizley et al., 2007; Bizley and King, 2008; rat 17%: Wallace et al., 2004; prairie vole 13%: Campi et al., 2007; macaque monkey 12%–59%: Brosch et al., 2005;Kayser et al., 2009).

Our present results do also show very specific exceptions to the general pattern of morphological change. For example, spine number and density of apical dendrites increase in S1-HL following auditory and somatosensory deprivation. Such an increase in spine density was also observed in S1-BF (barrel cortex) of permanently whisker-trimmed mice at P30 (Chen et al., 2015). One reason for this exception could be the early onset of somatosensation (Fuller and Wimer, 1966) and a more mature developmental stage of the intercortical somatosensory system at the time point of deprivation compared to the other sensory systems. It would also be interesting for future studies on gerbils whether neurons in S1-HL (with potentially numerous multisensory intercortical inputs) and neurons in S1-BF (with virtually no multisensory intercortical inputs) are differentially affected by early somatosensory deprivation.

There are also other changes in the dendritic branching patterns of supragranular layer III pyramidal neurons in A1, V1, and S1-HL, which seem to be more areal-specific. Generally, the branching of apical and basal dendrites, as seen by the number of intersections and dendritic lengths, was increased in all three areas following visual and somatosensory deprivation, whereas dendritic branching, particularly of apical dendrites, was decreased in all three areas following auditory deprivation. Since this decrease mainly affected apical dendrites one may speculate that it is related to unique changes in intercortical connectivity (number of connections, layer-specific feedforward/feedback characteristics) specifically following auditory deprivation. However, such unique changes in the nature of intercortical connections have not been seen previously (Henschke et al., 2018a); thus, further investigations are needed on this topic.

### Some Speculations About Further Development

Our study may also provide a bridge towards findings in long-term deprived adult animals and human subjects (Figure 11C). Despite some variability in the reports, adult animals with early loss of sensory experience show usually increased spine densities along dendrites of supragranular pyramidal neurons in primary cortical areas of the deprived sensory modality (A1, early deaf cats: Clemo et al., 2017; S1, early whisker-trimmed mice: Chen et al., 2015). Interestingly, spine density in cortical areas with more multisensory inputs like field FAES (auditory field of the anterior ectosylvian sulcus) was also increased (early deaf cats: Clemo et al., 2016). Dendritic branching in primary areas is not considerably different between deprived and normal animals (S1: Schubert et al., 2013; A1: Heumann and Rabinowicz, 1982; McMullen and Glaser, 1988; Clemo et al., 2017; V1: Heumann and Rabinowicz, 1982). Also, only subtle changes of sensory matched and non-matched connections of V1 and A1 were detected in adult animals following, for example, bilateral neonatal enucleation in mice (Charbonneau et al., 2012) and early deafening (Sanchez-Vives et al., 2006; Meredith and Allman, 2012; Chabot et al., 2015) or congenital deafness (Barone et al., 2013) in cats and ferrets. Thus, we speculate that during adolescence of early sensory deprived individuals, exuberant axonal projections from both sensory matched and non-matched sources, which were formed at the end of the sensory critical period due to “non-reliable” alterations of the sensory inputs, are retracted, leading to a similar number of crossmodal and other connections (i.e., retrogradely labeled input neurons) in deprived and non-deprived adults. At the same time, synaptic contacts, which were pruned at the end of the critical period due to the “non-reliable” sensory inputs, are newly formed in order to establish “reliable” synaptic contacts underlying crossmodal (compensatory) plasticity effects like enhanced activity to non-matched sensory stimuli and enhanced functional connectivity (for Refs. see below).

Several other functional-anatomical factors than morphological alterations on supragranular pyramidal neurons might additionally contribute to the above described developmental scenario during adolescence. These include changes in local inhibitory circuitry (e.g., increased lateral inhibition: Petrus et al., 2015; Nakajima et al., 2016) and thalamocortical circuitry (e.g., strengthened thalamocortical transmission: Yu et al., 2012; Petrus et al., 2014) as well as effects on other excitatory cell types (i.e., granular and subgranular pyramidal and non-pyramidal spiny neurons). On these cells, for example, spine density was found to be either unchanged (apical dendrites of layer V pyramidal neurons in A1 of early blind mice: Heumann and Rabinowicz, 1982; basal dendrites of subgranular pyramidal neurons in S1 of transiently whisker-trimmed P60 and P90 mice: Briner et al., 2010; Chen et al., 2015; apical and basal dendrites of subgranular pyramidal neurons in A1 and FAES of early deaf cats: Clemo et al., 2016, 2017) or reduced (apical dendrites of layer V pyramidal neurons in V1 of early blind mice: Valverde, 1968; Heumann and Rabinowicz, 1982; spiny non-pyramidal neurons in A1 of early deaf cats: Clemo et al., 2017). Differences between these studies may be due to different hierarchical levels of the investigated cortical areas (i.e., primary vs. higher order sensory area), the nature of the investigated modality (sensory deprived/matched vs. spared/non-matched), the timing of the deprivations (short-term vs. long-term, transient vs. permanent; young vs. adult), or species differences (e.g., developmentally, behaviorally) and have to be ruled out in future studies. Most importantly, there is a need to disentangle the interplay between the various cell types within the cortical layers and in particular with respect to their specific inputs (sensory matched, non-matched, other) as already discussed in the previous section.

At the functional level, the anatomical alterations during adolescence may lead to a higher neuronal activity, in particular in response to non-matched stimuli in spared cortical areas of adult individuals, as seen in all species investigated so far (e.g., opossum: Kahn and Krubitzer, 2002; hamster: Izraeli et al., 2002; mouse: Teichert and Bolz, 2017; rat: Piche et al., 2007; ferret: Meredith and Allman, 2012; cat: Rauschecker and Korte, 1993; human: Sadato et al., 1996). There is also a stronger functional connectivity between sensory processing cortices in adults with long-term sensory deprivation compared to normal individuals. For example, human studies revealed a stronger functional connectivity between the auditory, visual, and/or somatosensory in early deaf [electroencephalography: Sinke et al., 2019; functional magnetic resonance imaging (fMRI): Shiell et al., 2015; Bola et al., 2017] and blind people (resting-state fMRI: Pelland et al., 2017; dynamic causal modeling (DCM) of fMRI data: Collignon et al., 2013) and between V1 and S1 in Braille reading blinds (rsfMRI: Liu et al., 2007; DCM: Fujii et al., 2009).

At the molecular level, the enhanced crossmodal activity and functional connectivity in adults (as well as in young individuals) with early sensory loss may be mediated by various forms of synaptic strengthening and remodeling (for review, see Tropea et al., 2009; Lee and Whitt, 2015; Bridi et al., 2018). For example, visual deprivation drives AMPA receptors *via* extracellular serotonin into synapses of supragranular pyramidal neurons in rat barrel cortex (Jitsuki et al., 2011; Nakajima et al., 2016) and changes the AMPA receptor subunit (GluR1/R2) composition in rat V1 and S1 (Goel et al., 2006), leading to a specific synaptic strengthening in these areas. Likewise, deafening causes a N-Methyl-D-aspartate (NMDA) receptor activation and consequent long-term potentiation at synapses in adult mice V1 (Rodríguez et al., 2018).

For future studies, the ideal would be to test the relationship between morphological, connectional, and functional features of neurons in normal and deprived animals at different ages directly. Such an approach would require, for example, (intra-) cellular recordings from neurons with identified long-range inputs before and after deprivation and their subsequent morphological investigation. Likewise, studies using pharmacological interventions would help to disentangle the interplay between molecular and functional-anatomical levels. Until then, we have to put information from available studies together in order to get a bigger picture about possible mechanisms during multisensory development as we attempted it here.

### Morphological Differences of Supragranular Pyramidal Neurons Across Primary Sensory Cortices and Their Functional Implications

To the best of our knowledge, our study provides the first morphological comparison of supragranular layer III pyramidal neurons between primary sensory cortices in a rodent species (Figure 4). In P28 gerbils, A1 neurons have generally most widely branched apical and basal dendrites with most spines and highest spine densities, which is particularly evident for the distal dendritic aspects. Taking the number and density of spines as an indicator for the number of synaptic inputs, this correlates with the finding from our previous study that A1 receives more numerous corticocortical (intercortical) and thalamocortical inputs from matched (auditory) and non-matched (visual, somatosensory) modalities than V1 and S1 (Henschke et al., 2018a). It also corresponds to results of a carnivore study (adult ferrets), where authors compared the morphology of (supra- and subgranular) pyramidal neurons in A1 and S1 and demonstrated consistently higher spine numbers and densities for A1 neurons (Clemo and Meredith, 2012). However, this finding was not always statistically significant and also lengths of apical and basal dendrites did not differ between A1 and S1 in adult ferrets (Clemo and Meredith, 2012).

In P28 gerbils, layer III pyramidal neurons in S1-HL and V1 have generally longer proximal dendrites with higher spine densities than A1 neurons. Since largest and most influential post-synaptic potentials arise from synapses on most proximal dendritic segments (Thomson and Deuchars, 1997; Bannister, 2005), inputs close to the soma of S1-HL and V1 neurons may be of other importance than for A1 neurons. In turn, A1 layer III pyramidal have more elaborated distal dendrites (see above). The functional reasons and consequences for these differences in proximal and distal dendritic processing between supragranular pyramidal neurons in A1, S1, and V1 are unclear so far, one can only speculate that they may relate to a higher demand on temporal precision and thus more refined spatio-temporal integration in the dendritic tress in the auditory domain, for example, necessary in sound localization (Chadderton et al., 2009).

We also showed that in P28 gerbils, S1-HL supragranular layer III pyramidal neurons have longer distal apical dendrites than V1 and A1 neurons, whereas A1 neurons have longest distal basal dendrites. This may reflect differences in the balance between cortical (mainly to apical dendrites) and thalamic (mainly to basal dendrites) inputs into layer III pyramidal neurons of these three primary areas. Again, the functional background of this morphological difference is unknown; however, due to the nearly absence of spiny stellate thalamorecipient neurons in layer IV of the A1 compared to the V1 and S1 (Smith and Populin, 2001; Staiger et al., 2004; da Costa and Martin, 2011) it was concluded that layer IIIb pyramidal neurons in A1 are the main recipients of thalamocortical inputs (Winer, 2011). Thus, longer distal basal dendrites may reflect this specific function.

Like in our study, pyramidal neurons in presumptive S1 of manatees showed also a greater dendritic branching (dendritic segment count) than in V1 (A1 was not analyzed). Unfortunately, authors did not distinguish between pyramidal neurons of different layers, between apical and basal dendrites, and their proximal and distal aspects (Reyes et al., 2015). Thus, there may be several commonalities in the areal-specific dendritic morphology of supragranular pyramidal neurons across species but also considerable species-specific differences, which have to be disentangled in future studies (see also DeFelipe et al., 2002).

## Conclusion

At the end of the critical sensory period, the loss of early sensory experience induces an increase of multisensory (and sensory matched) intercortical and thalamocortical connections of primary sensory areas presumably *via* axonal sprouting (Henschke et al., 2018a; Figures 11A,B). Generally, there are also more extensively branched dendrites of their post-synaptic target cells (supragranular pyramidal neurons) but their actual synaptic contacts (spines) are pruned (present study). This may lead to a reduced stimulus-evoked activity in the deprived and spared cortical areas. The higher correlated activity (i.e., stronger functional connectivity) between these areas (Henschke et al., 2018a) may be due to synaptic strengthening at the molecular and ultrastructural level (Bridi et al., 2018). We hypothesize that during adolescence, dendritic branches of supragranular pyramidal neurons, as well as axonal branches of input neurons, are pruned back to normal, but new synaptic contacts are formed and strengthened (Rodríguez et al., 2018; Figure 11C). This may lead to a higher stimulus-induced activity, particularly to non-matched sensory stimuli in spared areas (Meredith and Lomber, 2017), and a higher functional connectivity between primary sensory cortical areas in early deprived adults compared to individuals with normal sensory experience (Shiell et al., 2015).

## Data Availability Statement

The datasets generated for this study are available on request to the corresponding author.

## Ethics Statement

The animal study was reviewed and approved by the animal care committee of Sachsen-Anhalt, Germany (number of proposal for animal experimentation: 42502-2-1324 LIN).

## Author Contributions

EB and JH: conceptualization, writing—original draft. JH and TM: investigation. EB, FO, HS, JH, MB, and TM: writing—review and editing. EB, FO, and HS: funding acquisition.

## Conflict of Interest

The authors declare that the research was conducted in the absence of any commercial or financial relationships that could be construed as a potential conflict of interest.
